# Blockade of spinal dopamine D1/D2 receptor heteromers by *levo*-Corydalmine suppressed calcium signaling cascade in spinal neurons to alleviate bone cancer pain in rats

**DOI:** 10.7150/jca.91129

**Published:** 2024-01-01

**Authors:** Xiao-Nan Ma, Chang-Heng Yao, Yu-Jie Yang, Xue Li, Meng-Yuan Zhou, Jin Yang, Shen Zhang, Bo-Yang Yu, Wen-Ling Dai, Ji-Hua Liu

**Affiliations:** 1 The Public Laboratory Platform, China Pharmaceutical University, Nanjing, Jiangsu 211198, China.; 2 Jiangsu Key Laboratory of TCM Evaluation and Translational Research, School of Traditional Chinese Pharmacy, China Pharmaceutical University, Nanjing, Jiangsu 211198, China.; 3 State Key Laboratory of Natural Medicines, School of Traditional Chinese Pharmacy, China Pharmaceutical University, Nanjing, Jiangsu 210009, China.

**Keywords:** Dopamine D1 receptors, Dopamine D2 receptors, D1/D2DR heteromers, calcium signaling cascade, bone cancer pain, *levo*-Corydalmine

## Abstract

**Background:** Dopamine receptors have been reported to be involved in pain, while the exact effects and mechanism in bone cancer pain have not been fully explored.

**Methods:** Bone cancer pain model was created by implanting walker 256 mammary gland carcinoma into right tibia bone cavity. Primary cultured spinal neurons were used for *in vitro* evaluation. FLIPR, western-blot, immunofluorescence, and Co-IP were used to detect cell signaling pathway.

**Results:** Our results indicated that spinal dopamine D1 receptor (D1DR) and spinal dopamine D2 receptor (D2DR) could form heteromers in TCI rats, and antagonizing spinal D1DR and D2DR reduced heteromers formation and alleviated TCI-induced bone cancer pain. Further results indicated that D1DR or D2DR antagonist induced antinociception in TCI rats could be reversed by D1DR, D2DR, and D1/D2DR heteromer agonists. And Gq, IP3, and PLC inhibitors also attenuated TCI-induced bone cancer pain. *In vitro* results indicated that D1DR or D2DR antagonist decreased the Ca^2+^ oscillations upregulated by D1DR, D2DR, and D1/D2DR heteromer agonists in activated primary cultured spinal neurons. Moreover, inhibition of D1/D2DR heteromers induced antinociception in TCI rats was partially mediated by the CaMKII and MAPKs pathway. In addition, a natural compound *levo*-Corydalmine (*l-*CDL), could inhibit D1/D2DR heteromers and attenuate bone cancer pain.

**Results:** Inhibition of spinal D1/D2DR heteromers via *l-*CDL decreases excitability in spinal neurons, which might present new therapeutic strategy for bone cancer pain.

## Introduction

Common tumors, such as breast, lung and prostate cancer frequently metastasize to multiple bones in the body and induce significant bone pain. The mechanisms of bone cancer pain are highly complicated. Cancer cells metastasize to the bone where they release algogenic substances, protons, and acidosis acting on the receptors of peripheral nociceptors 1, 2 and thereby inducing peripheral sensitization. The spinal cord receives the input from the primary afferent neurons. The activated spinal neurons can in turn release various excitatory neurotransmitters including glutamate, adenosine triphosphate (ATP) and calcitonin gene-related peptide (CGRP) to act on their receptors on postsynaptic neurons, which can increase intracellar Ca^2+^ levels 3, 4. Upregulation of Ca^2+^ facilitates signal transmission by activating Ca^2+^-sensitive proteins, such as calcium/calmodulin-dependent protein kinase (CaMKII) and mitogen-activated protein kinase (MAPK) to further enhance excitability in spinal cord neurons.

The detailed role and mechanisms of D1DR or D2DR in chronic bone cancer pain largely remains unexplored. D1DR and D2DR were reported to be mainly expressed in neurons of the spinal cord 5, 6. Activated spinal neurons plays an important role in the development and maintenance of chronic pain 7. Inhibiting the activation and excitability of spinal neurons could markedly attenuate bone cancer pain 8. It has been reported that D1DR couples to Gs/olf proteins to activate cyclic adenosine monophosphate (cAMP) and D2DR couples to Gi/o proteins to inhibit adenylcyclase (AC) 9. It has been widely accepted that activated cAMP signaling can phosphorylate cAMP response element-binding protein (CREB) to increase neuronal excitability in rodent hippocampal neurons 10 and striatal neurons 11, while inhibiting cAMP decreases neuronal excitability 12. The dopamine D1/D2DR heteromers was first identified in rat striatum 13, and was reported to be coupled to the Gq/11 protein, a finding that suggested a direct link between dopamine and calcium signaling 14-18. Increased intracellular calcium has been implicated in the increased excitability of neurons 19 and the development of chronic pain. Our previous research indicated that spinal dopamine D1 and D2 receptor form heteromers in spinal cord in neuropathic pain 20. Herein, we further tested the hypothesis that D1DR and D2DR might form heteromers to induce the activation of spinal neurons thereby promoting the development of bone cancer pain. This study aimed to investigate the role and mechanisms of spinal D1DR, D2DR, and their heteromers in bone cancer pain.

*Corydalis yanhusuo* W.T. Wang is one of the most famous analgesic is China, and tetrahydroproberberines were the main active ingredients and has been shown to act as dopamine agonist or antagonist 21, 22. *l-*CDL, one of the trace tetrahydroproberberines of *Corydalis yanhusuo* W.T. Wang has been reported to exert strong anti-nociception without notable side effects 23-26. Our previous researches showed that *l-*CDL could inhibit the formation of D1/D2DR complex to alleviate neuropathic pain 20, so the present study further investigated whether it could inhibit the D1/D2DR complex in bone cancer pain relief.

## Materials and Methods

### Ethics Statement

All experimental protocols were approved by the Animal Experimentation Ethics Committee of China Pharmaceutical University and adhered to the guidelines of the International Association for the Study of Pain (IASP). Meanwhile, the experiments we did were designed to minimize suffering and the number of animals used.

### Experimental Animals

Sprague-Dawley rats weighing 180-220 g and 60-80 g were purchased from the Experimental Animal Center at Yangzhou University (Jiangsu Province, China, SCXK-SU-2016-0011). Rats were housed three per cage in a temperature and humidity-controlled environment on a 12 h light/dark cycle for 3-7 days to allow acclimatization. Rats were anesthetized with pentobarbital (50 mg/kg, i.p.) and euthanized with carbon dioxide. Subsequently, the rats were randomly allocated to the following groups: 1) Control; 2) TCI; 3) TCI + D1DR/D2DR antagonists (20 μg/20 μL, i.t.); 4) TCI + D1DR/D2DR siRNA (1 μg/20 μL, i.t.); 5) TCI + D1DR/D2DR/D1/D2DR heteromer agonists (2 μg/20 μL, i.t.); 6) TCI + D1DR/D2DR/D1/D2DR heteromer agonists (2 μg/20 μL, i.t.) + D1DR/D2DR antagonists group (20 μg/20 μL, i.t.); 7) TCI + *l*-CDL (15 mg/kg, p.o. or 15 μg/20 μL, i.t.); 8) TCI + D1DR/D2DR/D1/D2DR heteromer agonists (2 μg/20 μL, i.t.) + *l*-CDL (15 mg/kg, p.o. or 15 μg/20 μL, i.t.). Behavioral testing was performed during the light cycle (between 9:00 a.m. and 5:00 p.m.). Six animals were assigned to each group for behavioral test and four animals were assigned to each group for molecular testing.

### Materials

*levo*-Corydalmine (purity ≥ 99.0%, as detected by HPLC) was provided by China Pharmaceutical University (Nanjing, China). Anti-p-p44/42 MAPK (p-ERK1/2) (#4377S), anti-ERK1/2 (#4695S), anti-p-JNK (#9255S), anti-JNK (#9252S), anti-p-p38 MAPK (p-p38) (#9215S), anti-p38 MAPK (#9212S), anti-p-CaMKII (#12716S), and anti-CaMKII (#3362S) were purchased from Cell Signaling Technology (Beverly, MA). Anti-D1DR was purchased from Abcam (#ab20066) (Cambridge, MA) and Santa Cruz Biotechnology (#sc-31479) (Santa Cruz, CA). Anti-D2DR was from purchased Santa Cruz Biotechnology (Santa Cruz, CA) (#sc-5303). Neurobasal medium, fetal bovine serum and RPMI 1640 medium were purchased from Gibco (Gaithersburg, MD). Trypsin and soybean trypsin inhibitors were obtained from Atlanta Biologicals (Norcross, GA). Agonists and antagonists were purchased were from Tocris Bioscience (Ellisville, MO), NHS magnetic beads was purchased from the Enriching biotechnology (Nanjing, China), all other reagents were purchased from Sigma-Aldrich (St. Louis, MO).

The siRNA targeting D1DR (NM_012546) and D2DR (NM_012547) were synthesized by GenePharma Co. (Shanghai, China). The respective sequences were as follows, sense: A 5'-GGUGACCAACUUCUUUGUCTT-3', B 5'-GACAAAGAAGUUGGUCACCTT-3', antisense: A 5'-CUACUAUGCCAUGCUGCUCTT-3', B 5'-GAGCAGCAUGGCAUAGUAGTT-3'. Nonspecific oligonucleotide controls consisted in randomly scrambled sequences of siRNA groups (conRNA). 33 μg siRNA and 49.5 μg polyethyleneimine (PEI) was diluted in 165 μL of 5 % glucose solutions respectively and were mixed and incubated for 15 min at RT before use 27, 28. For the siRNA group, each rat received multiple daily intrathecal injections of D1DR and D2DR siRNA mixed solution (1 μg/20 μL) for 8 consecutive days, and the control RNA group receive conRNA (1 μg/20 μL) for 8 days. Antinociception was measured at 0.5 h after siRNA treatment for 1-7 day and at 0.5 h, 2 h, 4 h, and 8 h on the 8^th^ day after siRNA treatment.

### Model of Bone Cancer Pain induced by Intra-Tibia Inoculation of Walker 256 Mammary Gland Carcinoma Cells

TCI-induced bone cancer pain was established according to our previous research 29. Walker 256 mammary gland carcinoma cells (5×10^6^ cells/mL, 0.5 mL) were intraperitoneally injected into rats weighing 60-80 g. The ascites were extracted and centrifuged at 400 g for 6 min to get the cells 5-7 days later. The cells were washed with iced 0.01 M phosphate-buffered saline (PBS) and then diluted to a density of 1×10^5^ cells/μL with 0.01 M PBS. Rats were anesthetized and the tibia head of the left leg was exposed with minimal damage. Next, 5 μL Walker 256 ascites tumor cells were slowly injected into the medullary cavity, and 5 μL PBS were injected as a control. To stop the cells from coming out, the syringe was held still for 1 min and bone wax was subsequently applied for 3 min. The injection hole was closed with dental materials.

### Behavioral Assays for Bone Cancer Related Pain

Before the test, rats were placed in individual transparent plastic mesh cage to accommodate the environment, then a series of Von Frey hairs (1.4-15.0 g) were used to stimulate the hind paw of rats with logarithmically incremental stiffness for about 6 s each. A positive response was defined as a quick withdrawal or licking of hind paw upon the stimulus. Whenever a positive response to a stimulus occurred, the next lower Von Frey hair was applied, and vise verse. Each rat was tested three more times and the applied force (g) was recorded. Then the average of the threshold was measured as mechanical withdrawal threshold (MWT) 29.

### Intrathecal Injection Procedure

The rat was placed in a prone position and the midpoint between the tips of the iliac crest was located. Using a stainless steel needle (30 gauge) by means of lumbar puncture at the intervertebral space of L4-5 or L5-6. The injection did not affect the baseline pain threshold of the rats and a proper injection would be accompanied by a tail flick 30.

### Primary Cultures of Spinal Neurons

The spinal cords of the embryos were removed aseptically on day 13 of gestation 31 and digested in 0.15 % typsin at 37 °C for 25 min. The cell suspension was centrifuged at 200 g for 4 min and then resuspended in solution containing DNase and soybean trypsin inhibitor. The solution containing MgCl_2_ and CaCl_2_ was added to the cells for 15 min, and the supernatant was collected and centrifuged at 200 g for 4 min. Neurobasal plating medium containing 10 % fetal bovine serum (FBS) supplement and 1 % *l-*glutamine was added. Cells were planted onto poly-*l-*lysine-pretreated 96-well (9 mm) clear-bottomed black plates with a density of 2.5×10^6^ cells/well 19, 32.

### Measurement of Intracellular Ca^2+^ Concentration

On day 9, the dye loading buffer containing 4 μM fluo-8 was added (100 μL/well) and incubated for 1 h. Then the cells were subsequently washed 5 times with Locke's buffer (the vehicle), leaving a final volume of 150 μL in each well. The plate was then transferred to a FLIPR (Molecular Devices, Sunnyvale, CA) chamber. Fluorescence reading was taken for 5 min to establish the baseline, and then the first test compound solutions (8 ×) (25 μL) were added to the corresponding well. 5 min after the fluorescence readings were taken for, the second compounds (25 μL) were added to the cells and the fluorescence readings were taken for another 10 min. The supernatant of primary culture spinal astrocytes stimulated with LPS for 12 h was added to the spinal neurons at the day 9 for 0.5 h before the measurement of Ca^2+^.

### Western Blotting

In brief, spinal cord segments at L4-L6 were collected at 2 h after drug treatment and lysed in RIPA. The supernatant was collected and separated on sodium dodecyl sulfate-polyacrylamide gels, and transferred onto polyvinylidene difluoride membranes. The membranes were blocked with 5 % bovine serum albumin (BSA) for 2 h at room temperature (RT) and incubated with primary antibodies for 3 h at RT and overnight in 4 ℃. Subsequently, the membranes were washed with 0.1 % tris buffered saline tween (TBST) and incubated with secondary antibodies (1:3000) at RT for 2 h. The immunoreactivity was detected using enhanced chemiluminescence (ECL) regents (PerkinElmer, Waltham, MA). Data were analyzed with the associated software Quantity one-4.6.5 (Bio-Rad Laboratories).

### Immunofluorescence

The L4-L6 spinal cords were collected after the rats were perfused with 0.01 M PBS followed by 4 % paraformaldehyde (PFA) on day 14 after the model was established. The spinal cords were post-fixed with the same 4 % (PFA) for 1 day and then transferred to 30 % sucrose for 3-5 days. The spinal cords were cut into 25 μm thick segments and blocked with 10 % normal donkey serum containing 0.3 % Triton-X-100 (Sigma-Aldrich, St. Louis, MO). Subsequently, the sections were incubated with the primary antibodies for 16-19 h at 4 ℃ and then incubated with secondary antibodies for 2 h after washed with 0.01 M PBS. The tissue sections were washed with PBS and mounted to be observed under a laser-scanning microscopy (Carl Zeiss LSM700, Germany). To obtain quantitative measurements, 8 images were evaluated for each group and photographed at the same exposure time to generate the raw data. Fluorescence intensities in the different groups were analyzed using Image Pro Plus 6.0 (Media Cybernetics, Silver Spring, MD, USA).

### Co-immunoprecipitation

In brief, 10 μg primary antibody was diluted with 500 μL coupling buffer (rat anti-D1DR or mouse anti-D2DR) and added to the NHS magnetic beads. After incubated sufficiently for 4 h at 4 ℃, the supernatant was removed and 500 μL blocking buffer was added to incubated for 1 h at 4 ℃. Tissues (spinal cord segments at L4-L6 of rats) were lysed in ice-cold RIPA buffer, and incubated with beads-Ab heteromers overnight at 4 ℃. After that, the immunoprecipitates were incubated with 100 μL elution buffers for 5 min at RT to dissociate the heteromers. The supernatant was transferred and incubated in SDS sample buffer for 10 min at 100 °C.

### Statistical Analysis

All values are depicted as mean ± SEM and the statistical analyses were performed using SPSS Rel 15 (SPSS Inc., Chicago, IL, USA). Data of western blot, immunofluorescence and behavioral tests were statistically analyzed by one-way analysis of variance (ANOVA) and two-way ANOVA followed by Bonferroni's post-hoc tests with significance at *P* < 0.05.

## Results

### Blockade of spinal dopamine D1DR and D2DR attenuated TCI-induced bone cancer pain and the expression of D1/D2DR heteromers were significantly increased in TCI-induced bone cancer pain

**Figure 1:**
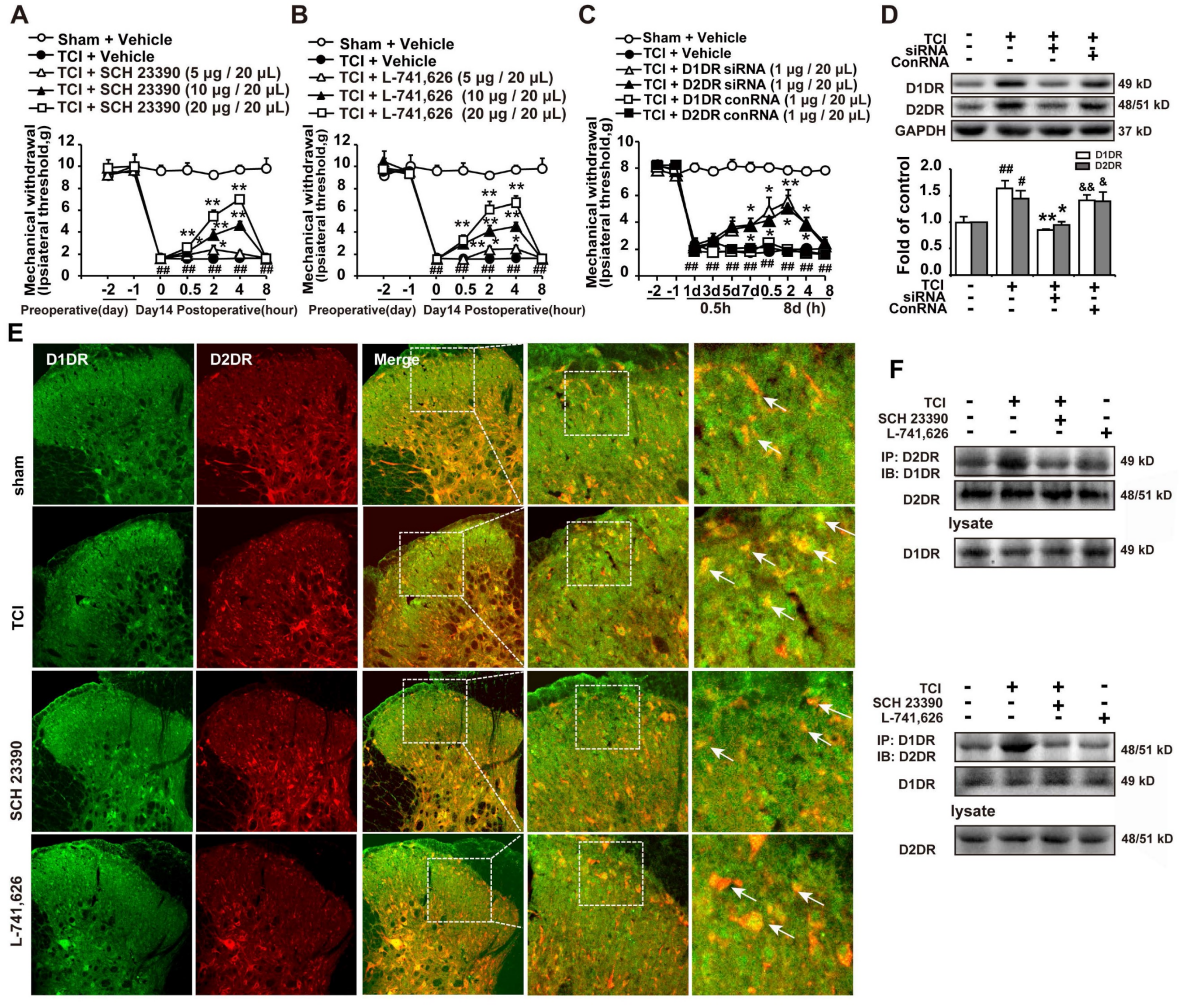
D1DR, D2DR form heteromers and antagonize spinal D1DR, D2DR alleviated pain in TCI rats. (A, B) Time course of the mechanical thresholds of SD rats after a single administration of D1DR antagonist SCH 23390 (5, 10, 20 μg/20 μL, i.t.) and D2DR antagonist L-741,626 (5, 10, 20 μg/20 μL, i.t.) in TCI rats on the 14^th^ day after the surgery (n = 6, *P < 0.05, **P < 0.01, compared with 0 h of each group; # *P* < 0.05, ##*P* < 0.01, compared with control group). (C) Time course of the mechanical thresholds of SD rats after a single administration of D1DR siRNA (1 μg/20 μL, i.t.) and D2DR siRNA (1 μg/20 μL, i.t.) in TCI rats on the 14^th^ day after the surgery (n = 6, *P < 0.05, **P < 0.01, compared with 0 h of each group; # *P* < 0.05, ##*P* < 0.01, compared with control group). (D) The expression of D1DR and D2DR after rats intrathecal administration of D1DR and D2DR siRNA (1 μg/20 μL, i.t.) for 8 days respectively (n = 4, # *P* < 0.05, ## *P* < 0.01, compared with control group, * *P* < 0.05, ** *P* < 0.01, compared with TCI group;). (E**)** Colocalization of D1DR (green) and D2DR (red) in the spinal cord of TCI rats after intrathecal administration of D1DR and D2DR antagonists. (F**)** Co-IP of D1DR and D2DR in the spinal cord of TCI rats after intrathecal administration of D1DR and D2DR antagonist.

Our results indicated that intrathecal administration of D1DR and D2DR antagonists (5, 10, and 20 μg/20 μL) significantly attenuated TCI-induced bone cancer (Figure 1A, B). Intrathecal administration of D1DR and D2DR siRNA (1 μg/20 μL) for 7 and 8 days respectively also alleviated TCI-induced chronic pain, while control RNA (conRNA) did not affect the mechanical threshold of rats (Figure 1C). Western blot results showed that in the siRNA group, the expression of D1DR and D2DR was decreased in the spinal cord, compared to that in the control (Figure 1D). Furthermore, the immunofluorescence (Figure 1E) and Co-IP (Figure 1F) results showed that D1DR and D2DR co-expressed in the spinal cord of rats. The co-expression of D1DR and D2DR was significantly upregulated in TCI-induced bone cancer pain. Intrathecal administration of D1DR and D2DR antagonists decreased D1DR and D2DR co-expression.

### Spinal D1DR and D2DR formed complexes to promote TCI-induced bone cancer pain through the Gq-PLC-IP3 pathway

**Figure 2:**
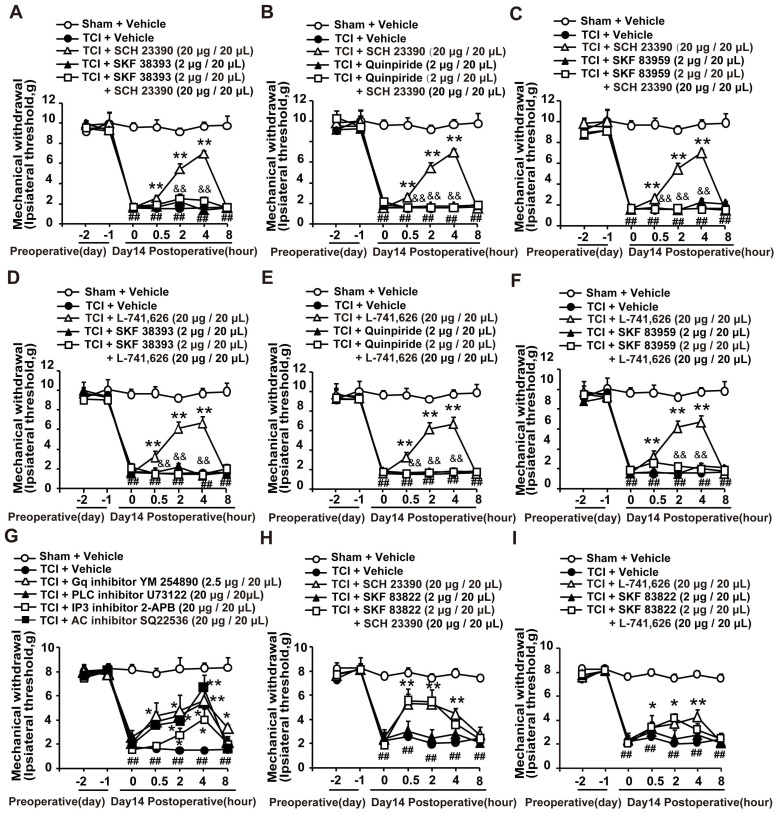
D1DR and D2DR antagonists induced antinociception might be mediated by D1/D2DR heteromers. (A, B, C) The mechanical thresholds of TCI rats after coadministration of D1DR agonist SKF 38393 (2 μg/20 μL, i.t.), D2DR agonist Quinpride (2 μg/20 μL, i.t.), D1/D2DR heteromer agonist SKF 83959 (2 μg/20 μL, i.t.) respectively with D1DR antagonist SCH 23390 (20 μg/20 μL, i.t.). (D, E, F) The mechanical thresholds of TCI rats after co-administration of SKF 38393 (2 μg/20 μL, i.t.), Quinpride (2 μg/20 μL, i.t.), and SKF 83959 (2 μg/20 μL, i.t.) respectively with D2DR antagonist L-741,626 (20 μg/20 μL, i.t.) (SKF 38393, Quinpride and SKF 83959 were administrated 15 min before SCH 23390 or L-741,626 administration). (G) Time course of the mechanical thresholds of SD rats after a single administration of AC inhibitor SQ22536 (20 μg/20 μL), Gq inhibitor YM 254890 (2.5 μg/20 μL), PLC inhibitor U73122 (20 μg/20 μL), and IP3 inhibitor 2-APB (20 μg/20 μL) in TCI rats on the 14th day after the surgery. (H, I) The mechanical thresholds of TCI rats after coadministration of D1DR agonist SKF 83822 (2 μg/20 μL, i.t.) with SCH 23390 or L-741,626 (20 μg/20 μL, i.t.) (SKF 83822 was administrated 15 min before SCH 23390 and L-741,626 treatment) (n = 6, *P < 0.05, **P < 0.01, compared with 0 h, #*P* < 0.05, ##*P* < 0.01, compared with control group, &*P* < 0.05, &&*P* < 0.01, compared with TCI + D1DR/D2DR antagonist group).

It has been reported that both D1DR and D2DR antagonists could reduce D1/D2DR heteromers 16, 33. Our results showed that the antinociception induced by D1DR antagonist SCH 23390 (Figure 2A-C) and D2DR antagonist L-741,626 (Figure 2D-F) could not only be reversed by D1DR agonist SKF 38393, but also alleviated by D2DR agonist Quinpiride, and D1/D2DR heteromer agonist SKF 83959. Furthermore, Gq inhibitor YM 254890, PLC inhibitor U73122, IP3 inhibitor 2-APB, and AC inhibitor SQ22536 could attenuate TCI-induced chronic bone cancer pain (Figure 2G). To explore whether D1DR antagonist-induced antinociception was mediated by cAMP, D1DR agonist SKF 83822, which exclusively activated the cAMP was used. Antinociception induced by D1DR antagonist SCH 23390 (Figure 2H) and D2DR antagonist L-741,626 (Figure 2I) could not be reversed by SKF 83822.

### D1DR, D2DR, and D1/D2DR agonists-upregulated Ca^2+^ oscillations in primary cultured spinal neurons could be inhibited by D1DR and D2DR antagonists

**Figure 3:**
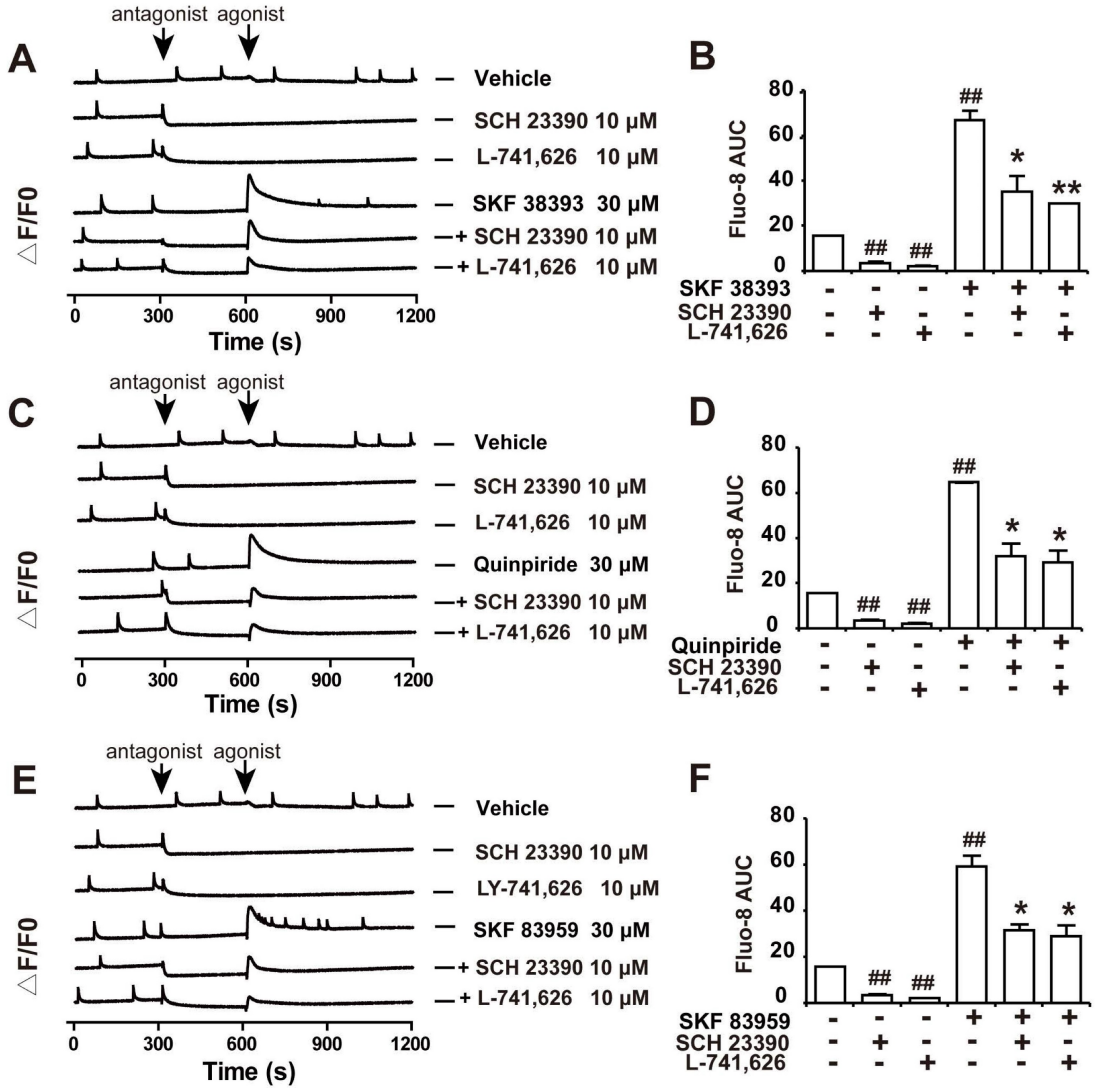
D1DR and D2DR antagonists inhibited D1DR, D2DR, D1/D2DR agonist upregulated Ca^2+^ oscillations in spinal neurons. (A, B) Representative traces and AUC of D1DR antagonist SCH 23390 (10 μM) (trace 2), D2DR antagonist L-741,626 (10 μM) (trace 3), D1DR agonist SKF 38393 (30 μM) (trace 4) on the Ca^2+^ oscillations and the effect of SCH 23390 (10 μM) and L-741,626 (10 μM) on SKF 38393 (30 μM) (trace 5, 6) induced rise in cytoplasmic Ca^2+^ in spinal neurons stimulated with the supernatant of primary astrocytes which stimulated with LPS (100 ng/ mL) for 12 h. (C, D) Representative traces and AUC of SCH 23390 (10 μM) (trace 2), L-741,626 (10 μM) (trace 3), D2DR agonist Quinpiride (30 μM) (trace 4) on the Ca^2+^ oscillations and the effect of SCH 23390 (10 μM), L-741,626 (10 μM) on Quinpiride (30 μM) (trace 5, 6) induced rise in cytoplasmic Ca^2+^ in spinal neurons stimulated with the supernatant of primary astrocytes which stimulated with LPS (100 ng/ mL) for 12 h (n = 3). (E, F) Representative traces and AUC of SCH 23390 (10 μM) (trace 2), L-741,626 (10 μM) (trace 3), D1/D2DR heteromer agonist SKF 83959 (30 μM) (trace 4) on the Ca^2+^ oscillations and the effect of SCH 23390 (10 μM) and L-741,626 (10 μM) on SKF 83959 (30 μM) (trace 5, 6) induced rise in cytoplasmic Ca^2+^ in spinal neurons stimulated with the supernatant of primary astrocytes which stimulated with LPS (100 ng/ mL) for 12 h (n = 3) (#*P* < 0.05, ##*P* < 0.01, compared with control group, &*P* < 0.05, &&*P* < 0.01, compared with agonist group).

Our results showed that both D1DR antagonist SCH 23390 and D2DR antagonist L-741,626 could eliminate the basal synchronous Ca^2+^ oscillations activity in primary cultured spinal neurons (Figure 3A and B, trace 2-3; time scale 300-600 seconds). Administration of D1DR agonist SKF 38393, D2DR agonist Quinpiride, and D1/D2DR heteromer agonist SKF 83959 could increase Ca^2+^ oscillations in spinal neurons (Figure 3A-F, trace 4; time scale 600-900 seconds), which could be eliminated with D1DR and D2DR antagonists, respectively (Figure 3A-F, trace 4-6; time scale 600-850 seconds). Herein, the supernatant of primary cultured spinal actrocytes stimulated with LPS for 12 h was added to the spinal neurons at the day 9 for 0.5 h.

### Intrathecal administration of D1DR and D2DR antagonists/siRNA inhibited the expression of p-CaMKII, p-ERK, p-JNK, and p-p38 in the spinal cord

**Figure 4:**
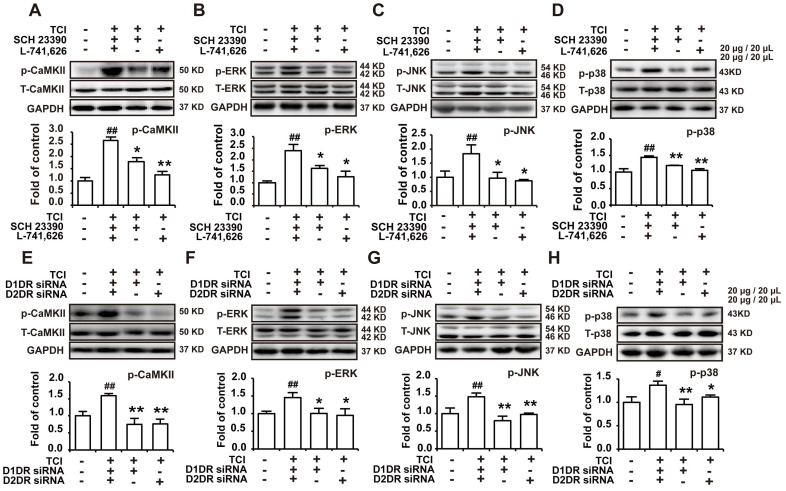
Antagonize spinal D1DR and D2DR inhibited the expression of p-CaMKII, p-MAPKs in the spinal cord. (A, B, C, D) The expression of p-CaMKII, p-ERK, p-JNK, and p-p38 in TCI rats after D1DR and D2DR antagonist (20 μg/20 μL, i.t.) was administrated. (E, F, G, H) The expression of p-CaMKII, p-ERK, p-JNK, and p-p38 in TCI rats after D1DR and D2DR siRNA (1 μg/20 μL, i.t.) was administrated. The L4-L6 spinal cord was collected 2 h after D1DR and D2DR antagonist/siRNA treatment (n = 4, #* P* < 0.05, ## *P* < 0.01, compared with control group, *P < 0.05, **P < 0.01, compared with TCI group).

D1/D2DR heteromers couple to Gq to increase intracellular Ca^2+^, which in turn activate several kinases including CaMKII and MAPKs 34. Our results herein indicated that D1DR and D2DR antagonists could decrease the upregulated expression of p-CaMKII, p-ERK, p-JNK, and p-p38 in the spinal cord (Figure 4A-D). And D1DR and D2DR siRNA also significantly decreased the expression of p-CaMKII, p-ERK, p-JNK, and p-p38 (Figure 4E-H).

### *l-*CDL induced antinociception in TCI rats could be reversed by D1DR, D2DR, and D1/D2DR heteromer agonists

**Figure 5:**
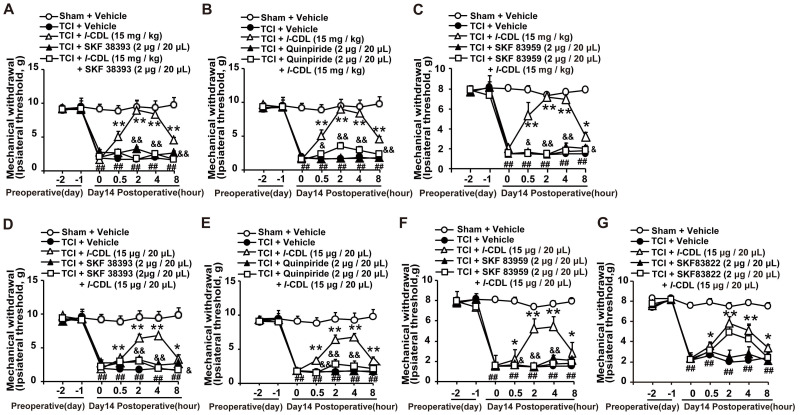
*l-*CDL induced antinociception in TCI rats was reversed by D1DR, D2DR and D1/D2DR heteromer agonist. (A, B, C) The mechanical thresholds of TCI rats after co-administration of D1DR agonist SKF 38393 (2 μg/20 μL, i.t.), D2DR agonist Quinpride (2 μg/20 μL, i.t.), D1/D2DR heteromer agonist SKF 83959 (2 μg/20 μL, i.t.) respectively with *l-*CDL (15 mg/kg, p.o). (D, E, F) The mechanical thresholds of TCI rats after co-administration of SKF 38393 (2 μg/20 μL, i.t.), Quinpride (2 μg/20 μL, i.t.), and SKF 83959 (2 μg/20 μL, i.t.) respectively with *l-*CDL (15 μg/20 μL, i.t.). (G) The mechanical thresholds of TCI rats after co-administration of D1DR agonist SKF 83822 (2 μg/20 μL, i.t.) with *l-*CDL (15 μg/20 μL, i.t.) (SKF 38393, Quinpride and SKF 83959 and SKF 83822 was administrated 15 min before *l-*CDL treatment) (n = 6, *P < 0.05, **P < 0.01, compared with 0 h, #* P* < 0.05, ##*P* < 0.01, compared with control group, &*P* < 0.05, &&*P* < 0.01, compared with TCI +* l-*CDL group).

Our study showed that *l-*CDL showed high affinity to D1DR (see Additional file 1: Figure s1) and D2DR 35 with a half maximal inhibitory concentration (IC50) of 0.20 μM and 0.86 μM, respectively. Herein, our results indicated that D1DR agonist SKF 38393, D2DR agonist Quinpiride, and D1/D2DR heteromer agonist SKF 83959 could reverse both intragastic (15 mg/kg) (Figure 5A-C) and intrathecal (15 μg/20 μL) (Figure 5D-F) administration of *l-*CDL induced antinociception in TCI rats (SKF 38393, Quinpiride, and SKF 83959 were administrated 15 min before* l-*CDL treatment). However, *l-*CDL (15 μg/20 μL, i.t.) induced antinociception could not be reversed by SKF 83822 (Figure 5G).

### *l-*CDL induced inhibition of p-CaMKII, p-ERK, p-JNK, and p-p38 could be reverse by D1DR, D2DR, and D1/D2DR heteromer agonists in the spinal cord of TCI rats

**Figure 6:**
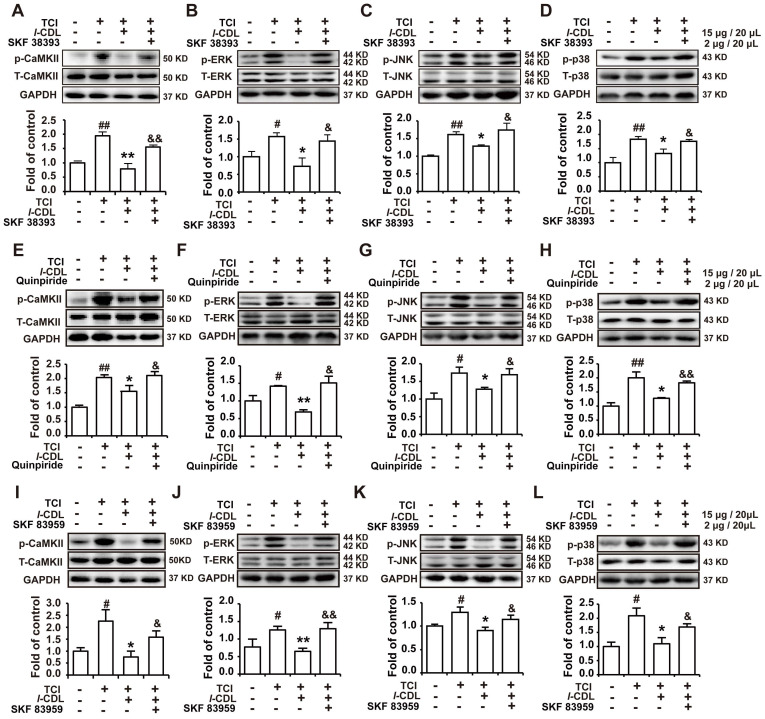
D1DR, D2DR, and D1/D2DR heteromer agonist reversed *l-*CDL induced inhibition of spinal p-CaMKII and p-MAPKs. (A, B, C, D) The expression of p-CaMKII, p-ERK, p-JNK, and p-p38 in TCI rats after co-administration of D1DR agonist SKF 38393 (2 μg/20 μL, i.t.) and* l-*CDL (15 mg/kg, p.o.). (E, F, G, H) The expression of p-CaMKII, p-ERK, p-JNK and p-p38 in TCI rats after co-administration of D2DR agonist Quinpiride (2 μg/20 μL, i.t.) and* l-*CDL (15 mg/kg, p.o.). (I, J, K, L) The expression of p-CaMKII, p-ERK, p-JNK and p-p38 in TCI rats after co-administration of D1/D2DR heteromer agonist SKF 83959 (2 μg/20 μL, i.t.) and* l-*CDL (15 mg/kg, p.o.) (SKF 38393, Quinpiride, and SKF 83959 was administrated 15 min before *l-*CDL treatment), the L4-L6 spinal cord was collected 2 h after *l-*CDL treatment (n = 4, #*P* < 0.05, ##*P* < 0.01, compared with control group, *P < 0.05, **P < 0.01, compared with TCI group, $*P* < 0.05, $$*P* < 0.01, compared with TCI + *l-*CDL group).

Our previous study indicated that *l-*CDL markedly alleviated TCI-induced bone cancer pain. Herein, our results further confirmed that *l-*CDL (15 mg/kg, p.o.) could decrease the expression of p-CaMKII, p-ERK, p-JNK, and p-p38. Intrathecal administration of D1DR agonist SKF 38393 (Figure 6A-D), D2DR agonist Quinpiride (Figure 6E-H), and D1/D2DR heteromer agonist SKF 83959 (Figure 6I-L) (15 minutes before *l-*CDL on the 14th day after TCI) reversed *l-*CDL (15 μg/20 μL, i.t.) induced inhibition of upregulated p-CaMKII, p-ERK, p-JNK, and p-p38 in TCI-induced bone cancer pain. These results suggested that p-CaMKII, p-ERK, p-JNK, and p-p38 are involved in D1/D2DR heteromers mediated analgesia of *l-*CDL.

## Discussion

In this study the principal findings are as follows: (1) Intrathecal administration of D1DR/D2DR antagonists or siRNA could significantly alleviate TCI-induced bone cancer pain; (2) D1DR and D2DR form heteromers in spinal neurons that promote bone cancer pain by activating Gq protein and thereby increasing neuronal excitability, leading to the activation of CaMKII and MAPK signaling; (3) *l-*CDL, a natural compound could attenuate TCI-induced chronic bone cancer pain through inhibiting D1/D2DR heteromers.

Activating or antagonizing spinal D1DR and D2DR have been reported could inhibit the development of pain 36-39. And D1DR and D2DR have been reported to form heteromers in rat or monkey brain and have a potentially considerable influence in disorders such as drug addiction, schizophrenia and depression 15-18. Our previous research also confirmed that D1DR and D2DR form complex to promote neuropathic pain 20. Herein, further results indicated that D1DR and D2DR could form heteromers in the spinal cord, and that intrathecal administration of both D1DR and D2DR antagonists could inhibit the heteromers in TCI rats which was consistent with the previous findings that D1/D2DR heteromers mediated signaling could be attenuated by D1DR and D2DR antagonists, respectively 16, 33. D1DR and D2DR antagonists-induced antinociception could be alleviated by D1DR, D2DR, and D1/D2DR heteromer agonists, which indicated that D1DR and D2DR antagonists attenuate TCI-induced bone cancer through inhibiting D1/D2DR heteromers.

D1/D2DR heteromers were reported to couple to Gq, which might lead to intracellular calcium mobilization from IP3 receptor-sensitive stores through a cascade of events involving rapid translocation of Gq to plasma membrane and activation of PLC. Gq protein, IP3-mediated calcium signaling, and PLC 40, 41 have all been reported to mediate nociceptor sensitization. Their effects in bone cancer pain have not been explored, however. To further confirm whether D1DR and D2DR antagonists attenuate TCI-induced bone cancer pain through inhibiting D1/D2DR heteromers with the subsequent suppression of the activation of the Gq-IP3-PLC pathway, the effects of Gq inhibitor YM254890, IP3 inhibitor 2-APB, and PLC inhibitor U73122 on bone cancer pain were explored. Intrathecal administration of YM 254890, 2-APB, and U73122 could all attenuate TCI-induced bone cancer pain. We also wondered whether D1DR and D2DR attenuated chronic bone cancer pain through Gi/o or Gs proteins. It has been reported that Gi/o protein inhibitor Pertussis toxin (PTX) produced hyperalgesia and allodynia 42. Herein, intrathecal administration of AC inhibitor SQ22536 could also attenuate TCI-induced bone cancer pain. In order to verify whether D1DR antagonist can attenuate TCI-induced bone cancer pain through the activation of AC through Gs protein, SKF 83822, which exclusively activates the cAMP 16, 43 was used. D1DR and D2DR antagonists-induced antinociception could not be reversed by SKF 83822 indicating that D1DR and D2DR antagonists-induced antinociception was not mediated through the inhibition of the AC pathway.

Further *in vitro* studies were conducted to confirm whether D1DR and D2DR form heteromers that increase the excitatory state of neurons. Spontaneous Ca^2+^ transients have been implicated in regulating plasticity in developing neurons 44. Primary cultured spinal neurons display synchronized spontaneous Ca^2+^ oscillations 19. Our study showed that administration of D1DR and D2DR antagonists could reduce the Ca^2+^ oscillations in spinal neurons. Administration of D1DR, D2DR, and D1/D2DR heteromer agonists increased Ca^2+^ oscillations, which in turn could be reduced by D1DR and D2DR antagonists. D1/D2DR heteromers couple to Gq, which activates CaMKII that in turn also promote the development of chronic pain through activating MAPK 34. Expression of p-CaMKII, p-ERK, p-JNK, and p-p38 was observed. Both D1DR, D2DR antagonists, and siRNA could also inhibit the expression of p-CaMKII, p-ERK, p-JNK, and p-p38.

The important role of D1/D2DR heteromers in TCI-induced bone cancer pain make them an attractive target to attenuate bone cancer pain. *l-*CDL, a trace ingredient from traditional Chinese medicine could significantly attenuate chronic bone cancer pain and other models of neuropathic pain in our previous studies without notable side effects 25, 26, 29. *l*-CDL belongs to the tetrahydroprotoberberines (THPBs), which have been reported to show affinity to dopamine receptors and possess a variety of beneficial effects without notable side effects 21, 22. *l-*CDL showed high affinities to both D1DR (see Additional file 1: Figure s1) and D2DR 35 with IC50 of 0.20 μM and 0.86 μM respectively. Herein, our results indicated that *l-*CDL-induced antinociception could be reversed by D1DR, D2DR, and D1/D2DR heteromer agonists but not SKF 83822, which exclusively activates the cAMP. *l-*CDL-induced inhibition of p-CaMKII, p-ERK, p-JNK, and p-p38 could be alleviated by D1DR, D2DR, and D1/D2DR heteromer agonists. *l*-CDL has been reported in our previous study to alleviate TCI-induced chronic bone cancer pain through inhibiting NMDA and mGlu1/5 receptors 29. Synergistic effects in such a multi-targets approach might largely explain the strong observed effects in attenuating bone cancer pain.

We provided the first experimental evidence that spinal D1DR and D2DR might promote chronic bone cancer pain through forming D1/D2DR heteromers, thereby leading to the activation of Gq proteins and the downstream CaMKII and MAPK signaling to increase excitability in spinal neurons. *l*-CDL, a natural compound, was found to attenuate TCI-induced chronic bone cancer pain by antagonizing spinal D1DR and D2DR to inhibit D1/D2DR heteromers, and in turn downstream CaMKII and MAPKs signaling. Altogether, the findings may provide new avenues to find more effective and safer targets for chronic bone cancer pain.

## Supplementary Material

Supplementary methods and figure.

## Abbreviations

D1DR: dopamine D1 receptors
D2DR: dopamine D2 receptors
TCI: tibia bone cavity tumor cell implantation
FLIPR: Fluorometric imaging plate reader
Co-IP: co-immunoprecipitation
ATP: adenosine triphosphate
CGRP: calcitonin gene-related peptide
CaMKII: calcium/calmodulin-dependent protein kinase
MAPK: mitogen-activated protein kinase
cAMP: cyclic adenosine monophosphate
AC: adenylcyclase
THPBs: tetrahydroprotoberberines
*l*-CDL: *levo*-Corydalmine
BSA: bovine serum albumin
TBST: tris buffered saline tween

## References

[1] Zheng XQ, Wu YH, Huang JF, Wu AM (2022). Neurophysiological mechanisms of cancer-induced bone pain. J Adv Res.

[2] Zajaczkowska R, Kocot-Kepska M, Leppert W, Wordliczek J (2019). Bone Pain in Cancer Patients: Mechanisms and Current Treatment. Int J Mol Sci.

[3] Finnerup NB, Kuner R, Jensen TS (2021). Neuropathic Pain: From Mechanisms to Treatment. Physiol Rev.

[4] Cao B, Scherrer G, Chen L (2022). Spinal cord retinoic acid receptor signaling gates mechanical hypersensitivity in neuropathic pain. Neuron.

[5] Zhu H, Clemens S, Sawchuk M, Hochman S (2007). Expression and distribution of all dopamine receptor subtypes (D(1)-D(5)) in the mouse lumbar spinal cord: a real-time polymerase chain reaction and non-autoradiographic in situ hybridization study. Neuroscience.

[6] Dai WL, Xiong F, Yan B, Cao ZY, Liu WT, Liu JH (2016). Blockade of neuronal dopamine D2 receptor attenuates morphine tolerance in mice spinal cord. Sci Rep.

[7] Wang K, Wang S, Chen Y, Wu D, Hu X, Lu Y (2021). Single-cell transcriptomic analysis of somatosensory neurons uncovers temporal development of neuropathic pain. Cell Res.

[8] Cai J, Fang D, Liu XD, Li S, Ren J, Xing GG (2015). Suppression of KCNQ/M (Kv7) potassium channels in the spinal cord contributes to the sensitization of dorsal horn WDR neurons and pain hypersensitivity in a rat model of bone cancer pain. Oncol Rep.

[9] Beaulieu JM, Gainetdinov RR (2011). The physiology, signaling, and pharmacology of dopamine receptors. Pharmacol Rev.

[10] Gruart A, Benito E, Delgado-Garcia JM, Barco A (2012). Enhanced cAMP response element-binding protein activity increases neuronal excitability, hippocampal long-term potentiation, and classical eyeblink conditioning in alert behaving mice. J Neurosci.

[11] Threlfell S, West AR (2013). Review: Modulation of striatal neuron activity by cyclic nucleotide signaling and phosphodiesterase inhibition. Basal Ganglia.

[12] Jancic D, Lopez de Armentia M, Valor LM, Olivares R, Barco A (2009). Inhibition of cAMP response element-binding protein reduces neuronal excitability and plasticity, and triggers neurodegeneration. Cereb Cortex.

[13] Lee SP, So CH, Rashid AJ, Varghese G, Cheng R, Lanca AJ (2004). Dopamine D1 and D2 receptor Co-activation generates a novel phospholipase C-mediated calcium signal. J Biol Chem.

[14] Perreault ML, Hasbi A, O'Dowd BF, George SR (2014). Heteromeric dopamine receptor signaling complexes: emerging neurobiology and disease relevance. Neuropsychopharmacology.

[15] Hasbi A, O'Dowd BF, George SR (2011). Dopamine D1-D2 receptor heteromer signaling pathway in the brain: emerging physiological relevance. Mol Brain.

[16] Hasbi A, Fan T, Alijaniaram M, Nguyen T, Perreault ML, O'Dowd BF (2009). Calcium signaling cascade links dopamine D1-D2 receptor heteromer to striatal BDNF production and neuronal growth. Proc Natl Acad Sci U S A.

[17] Calabresi P, Picconi B, Tozzi A, Ghiglieri V, Di Filippo M (2014). Direct and indirect pathways of basal ganglia: a critical reappraisal. Nat Neurosci.

[18] Hasbi A, Perreault ML, Shen MYF, Fan T, Nguyen T, Alijaniaram M (2017). Activation of Dopamine D1-D2 Receptor Complex Attenuates Cocaine Reward and Reinstatement of Cocaine-Seeking through Inhibition of DARPP-32, ERK, and DeltaFosB. Front Pharmacol.

[19] Kelamangalath L, Dravid SM, George J, Aldrich JV, Murray TF (2011). kappa-Opioid receptor inhibition of calcium oscillations in spinal cord neurons. Mol Pharmacol.

[20] Bao YN, Dai WL, Fan JF, Ma B, Li SS, Zhao WL (2021). The dopamine D1-D2DR complex in the rat spinal cord promotes neuropathic pain by increasing neuronal excitability after chronic constriction injury. Exp Mol Med.

[21] Parraga J, Cabedo N, Andujar S, Piqueras L, Moreno L, Galan A (2013). 2,3,9- and 2,3,11-trisubstituted tetrahydroprotoberberines as D2 dopaminergic ligands. Eur J Med Chem.

[22] Sun H, Zhu L, Yang H, Qian W, Guo L, Zhou S (2013). Asymmetric total synthesis and identification of tetrahydroprotoberberine derivatives as new antipsychotic agents possessing a dopamine D(1), D(2) and serotonin 5-HT(1A) multi-action profile. Bioorg Med Chem.

[23] Dai WL, Bao YN, Fan JF, Li SS, Zhao WL, Yu BY (2020). Levo-corydalmine attenuates microglia activation and neuropathic pain by suppressing ASK1-p38 MAPK/NF-kappaB signaling pathways in rat spinal cord. Reg Anesth Pain Med.

[24] Dai WL, Yan B, Bao YN, Fan JF, Liu JH (2020). Suppression of peripheral NGF attenuates neuropathic pain induced by chronic constriction injury through the TAK1-MAPK/NF-kappaB signaling pathways. Cell Commun Signal.

[25] Hu Y, Kodithuwakku ND, Zhou L, Li C, Han D, Fang W (2017). Levo-Corydalmine Alleviates Neuropathic Cancer Pain Induced by Tumor Compression via the CCL2/CCR2 Pathway. Molecules.

[26] Zhou L, Hu Y, Li C, Yan Y, Ao L, Yu B (2018). Levo-corydalmine alleviates vincristine-induced neuropathic pain in mice by inhibiting an NF-kappa B-dependent CXCL1/CXCR2 signaling pathway. Neuropharmacology.

[27] Njoo C, Heinl C, Kuner R In Vivo SiRNA Transfection and Gene Knockdown in Spinal Cord via Rapid Noninvasive Lumbar Intrathecal Injections in Mice. 2014(85):e51229.

[28] Gong N, Fan H, Ma AN, Xiao Q, Wang YX (2014). Geniposide and its iridoid analogs exhibit antinociception by acting at the spinal GLP-1 receptors. Neuropharmacology.

[29] Dai WL, Yan B, Jiang N, Wu JJ, Liu XF, Liu JH (2017). Simultaneous inhibition of NMDA and mGlu1/5 receptors by levo-corydalmine in rat spinal cord attenuates bone cancer pain. Int J Cancer.

[30] Wu XF, Liu WT, Liu YP, Huang ZJ, Zhang YK, Song XJ (2011). Reopening of ATP-sensitive potassium channels reduces neuropathic pain and regulates astroglial gap junctions in the rat spinal cord. Pain.

[31] Langlois SD, Morin S, Yam PT, Charron F (2010). Dissection and culture of commissural neurons from embryonic spinal cord. Journal of visualized experiments: JoVE.

[32] Cao Z, Cui Y, Busse E, Mehrotra S, Rainier JD, Murray TF (2014). Gambierol inhibition of voltage-gated potassium channels augments spontaneous Ca2+ oscillations in cerebrocortical neurons. The Journal of pharmacology and experimental therapeutics.

[33] Rashid AJ, So CH, Kong MM, Furtak T, El-Ghundi M, Cheng R (2007). D1-D2 dopamine receptor heterooligomers with unique pharmacology are coupled to rapid activation of Gq/11 in the striatum. Proc Natl Acad Sci U S A.

[34] Ji RR, Xu ZZ, Gao YJ (2014). Emerging targets in neuroinflammation-driven chronic pain. Nat Rev Drug Discov.

[35] Dai WL, Liu XT, Bao YN, Yan B, Jiang N, Yu BY (2018). Selective blockade of spinal D2DR by levo-corydalmine attenuates morphine tolerance via suppressing PI3K/Akt-MAPK signaling in a MOR-dependent manner. Exp Mol Med.

[36] Cobacho N, de la Calle JL, Paino CL (2014). Dopaminergic modulation of neuropathic pain: analgesia in rats by a D2-type receptor agonist. Brain research bulletin.

[37] Zhou J, Wang F, Xu C, Zhou Z, Zhang W (2017). KLF15 regulates dopamine D2 receptor and participates in mouse models of neuropathic pain. Biochemical and biophysical research communications.

[38] Rodgers HM, Yow J, Evans E, Clemens S, Brewer KL (2019). Dopamine D1 and D3 receptor modulators restore morphine analgesia and prevent opioid preference in a model of neuropathic pain. Neuroscience.

[39] Liu Y-Y, Wang T-X, Zhou J-C, Qu W-M, Huang Z-L (2019). Dopamine D1 and D2 receptors mediate analgesic and hypnotic effects of l-tetrahydropalmatine in a mouse neuropathic pain model. Psychopharmacology.

[40] Wirotanseng LN, Kuner R, Tappe-Theodor A (2013). Gq rather than G11 preferentially mediates nociceptor sensitization. Mol Pain.

[41] Galeotti N, Bartolini A, Ghelardini C (2003). The phospholipase C-IP3 pathway is involved in muscarinic antinociception. Neuropsychopharmacology.

[42] Womer DE, DeLapp NW, Shannon HE (1997). Intrathecal pertussis toxin produces hyperalgesia and allodynia in mice. Pain.

[43] Rashid AJ, O'Dowd BF, Verma V, George SR (2007). Neuronal Gq/11-coupled dopamine receptors: an uncharted role for dopamine. Trends Pharmacol Sci.

[44] Spitzer NC, Olson E, Gu X (1995). Spontaneous calcium transients regulate neuronal plasticity in developing neurons. J Neurobiol.

