# Supplementary material for: Blockade of spinal dopamine D1/D2 receptor heteromers by levo-Corydalmine suppressed calcium signaling cascade in spinal neurons to alleviate bone cancer pain in rats
Source: J Cancer. 2024 Jan 1;15(4):1041–52. doi: 10.7150/jca.91129 (PMC10788731; doi:10.7150/jca.91129)
Supplement: Supplementary file 1 — Supplementary methods and figure. [file jcav15p1041s1.pdf]

Blockade of spinal dopamine D1/D2 receptor heteromers by *levo*-Corydalmine suppressed calcium signaling cascade in spinal neurons to alleviate bone cancer pain in rats

Xiao-Nan Ma [1#], Chang-HengYao [2#], Yu-Jie Yang [2#], Xue Li [2#], Meng-Yuan Zhou[2], Jin Yang [2], Shen Zhang [2], Bo-Yang Yu [2,3], Wen-Ling Dai [2*], Ji-Hua Liu [2, 3*]

[1]*Jiangsu Key Laboratory of TCM Evaluation and Translational Research, School of* [21]

[2]*Jiangsu Key Laboratory of TCM Evaluation and Translational Research, School of Traditional Chinese Pharmacy, China Pharmaceutical University, Nanjing, Jiangsu 211198, China*

[3]*State Key Laboratory of Natural Medicines, School of Traditional Chinese Pharmacy, China Pharmaceutical University, Nanjing, Jiangsu 210009, China*

* Corresponding author: Ji-Hua Liu, Wen-Ling Dai

E-mail: liujihua@cpu.edu.cn; daiwenling@cpu.edu.cn

Tel: 0086-025-86185157

Fax: 0086-025-86185158

# These authors contributed equally to the work.

Additional file 1: Fig. s1. *l*-CDL shows micromolar affinity to D1DR with half

maximal inhibitory concentration (IC50) of 0.20 μM.

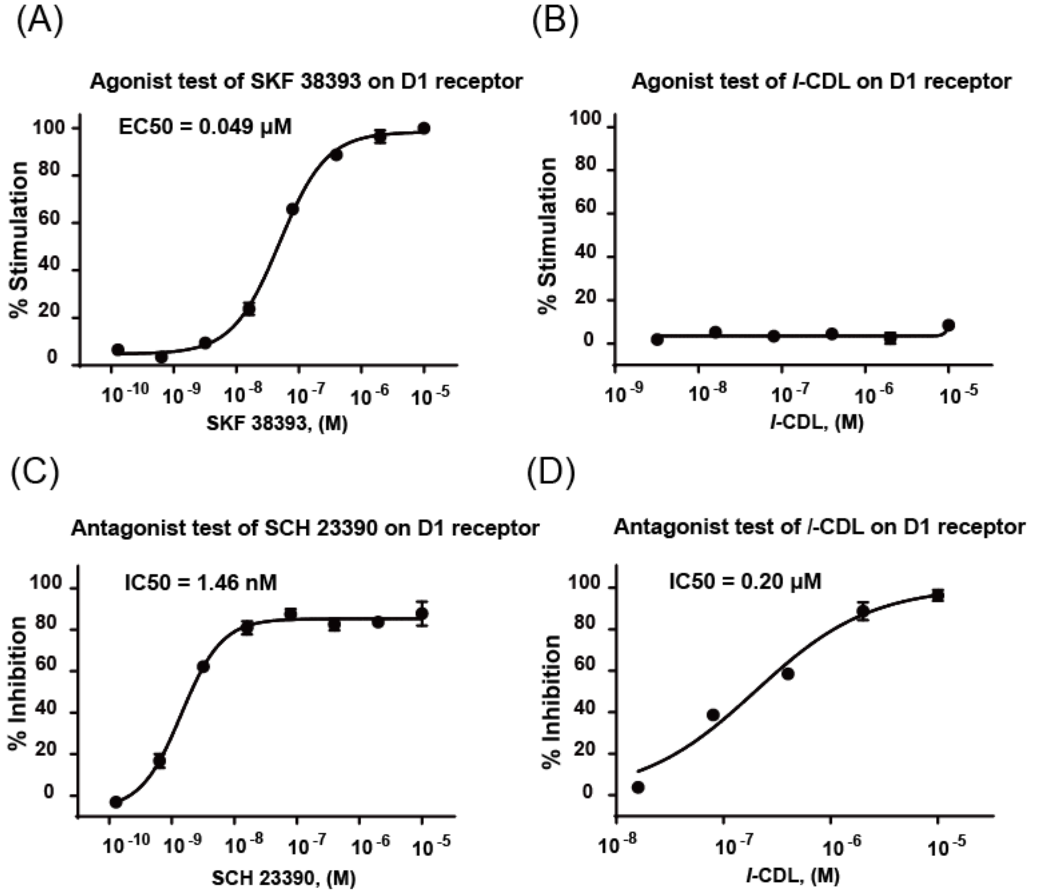

FIGURE s1 *l*-CDL shows micromolar affinity to D1DR with half maximal inhibitory concentration (IC50) of 0.20 μM. *l*-CDL could inhibit D1DR agonist SKF 38393 increased cAMP in D1/CHO-K1 cell lines with IC50 of 0.20 μM (Figure s1D), while it could not activate D1DR to increase cAMP (Figure s1B), indicating that *l*-CDL exhibited micromolar affinities to D1DR. The D1DR agonist SKF 38393 and D1DR antagonist SCH-23390 were used as positive control, SKF 38393 could increase cAMP in D1/CHO-K1 cell lines was half maximal effective concentration (EC50) of 0.049 μM ((Figure s1A), and D1DR antagonist SCH 23390 inhibited dopamine increased cAMP in D1/CHO-K1 cell lines with an IC50 of 1.46 nM (Figure s1C).

Additional experimental procedures

Cell culture

The D1/CHO-K1 cell lines were cultured in Ham's F12 containing 10% FBS supplement and 200 μg/mL Zeocin were added. The D1/CHO-K1 cell lines were plated onto 384 wells microplate with a density of 1.5×10^4 cells/well (20 μL/well). Cells were incubated at 37 °C with 5% CO_2 and 95% humidity for 18 hours.

cAMP analysis

The cAMP concentration was determined by cAMP direct immunoassay kit (Cisbio Bioassays). The The D1/CHO-K1 cells were planted onto a 384-well microplate with a density of 3000cell/5 μL. And 5 μL of the (2 ×) agonist or compound solutions were added to corresponding well to incubate for 30 min. Then the cells were incubated with 10 μL detection reagent for 1 h. The signals were detected at 665 nm and 620 nm. For agonist tests, *l*-CDL (2 ×) was added, for antagonist tests, *l*-CDL (1 ×) and agonist (1 ×) were added simultaneously.
